# Record‐High Thermoelectric Performance in Al‐Doped ZnO via Anderson Localization of Band Edge States

**DOI:** 10.1002/advs.202309291

**Published:** 2024-05-05

**Authors:** Illia Serhiienko, Andrei Novitskii, Fabian Garmroudi, Evgeny Kolesnikov, Evgenia Chernyshova, Tatyana Sviridova, Aleksei Bogach, Andrei Voronin, Hieu Duy Nguyen, Naoyuki Kawamoto, Ernst Bauer, Vladimir Khovaylo, Takao Mori

**Affiliations:** ^1^ WPI‐MANA, National Institute for Materials Science (NIMS) Tsukuba Ibaraki 305‐0044 Japan; ^2^ Graduate School of Pure and Applied Sciences University of Tsukuba Tsukuba Ibaraki 305‐8573 Japan; ^3^ Institute of Solid State Physics TU Wien Vienna A‐1040 Austria; ^4^ National University of Science and Technology MISIS Moscow 119049 Russia; ^5^ Prokhorov General Physics Institute of the Russian Academy of Sciences Moscow 119991 Russia; ^6^ Center for Basic Research on Materials (CBRM) National Institute for Materials Science (NIMS) Tsukuba Ibaraki 305‐0044 Japan; ^7^ Belgorod State University Belgorod 308015 Russia

**Keywords:** Anderson localization, chemical co‐precipitation, oxides, thermoelectric materials, wet chemistry, ZnO

## Abstract

Oxides are of interest for thermoelectrics due to their high thermal stability, chemical inertness, low cost, and eco‐friendly constituting elements. Here, adopting a unique synthesis route via chemical co‐precipitation at strongly alkaline conditions, one of the highest thermoelectric performances for ZnO ceramics ($PF_{\max}=$ 21.5 µW cm^−1^ K^−2^ and $zT_{\max}=$ 0.5 at 1100 K in ${\mathrm{Zn}}_{0.96}{\mathrm{Al}}_{0.04}O$) is achieved. These results are linked to a distinct modification of the electronic structure: charge carriers become trapped at the edge of the conduction band due to Anderson localization, evidenced by an anomalously low carrier mobility, and characteristic temperature and doping dependencies of charge transport. The bi‐dimensional optimization of doping and carrier localization enable a simultaneous improvement of the Seebeck coefficient and electrical conductivity, opening a novel pathway to advance ZnO thermoelectrics.

## Graphical abstract

This study reports on the thermoelectric properties of Al‐doped ZnO ceramics, synthesized via chemical co‐precipitation at strongly alkaline conditions. A large enhancement of the thermoelectric performance (zT = 0.5) is obtained and attributed to disorder‐induced modifications of the electronic structure, namely Anderson localization of band edge states, as evidenced by anomalous temperature and doping dependencies of electronic transport.

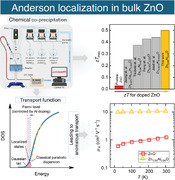

## 1 Introduction

Thermoelectric (TE) generators are able to convert waste heat into useful electrical energy due to the Seebeck effect. The conversion efficiency of such devices is directly related to material‐specific properties subsumed in the dimensionless figure of merit $zT=\alpha^{2}\sigma T/\kappa$, where α is the Seebeck coefficient, σ the electrical conductivity, T the absolute temperature and κ the thermal conductivity, including electron and lattice contributions $\kappa =\kappa_{\text{el}}+\kappa_{\text{lat}}$.^[^ ^1^ ^]^ The intricate interplay between electronic and thermal transport creates several trade‐offs, making the enhancement of zT one of the most formidable challenges in the realm of materials science. Nonetheless, the last decades of research have seen significant progress in the development of high‐performance TE materials such as chalcogenides,^[^ ^2^, ^3^, ^4^, ^5^, ^6^, ^7^, ^8^ ^]^ ${\mathrm{CoSb}}_{3}$‐based skutterudites,^[^ ^9^, ^10^ ^]^ and Half‐Heusler alloys.^[^ ^11^, ^12^, ^13^ ^]^ As a downside, most of these materials are easily decomposed and/or oxidized at high temperatures and in air atmosphere, presenting a major bottleneck for practical applications in the industry.

In this regard, oxides (e.g., oxyselenides,^[^ ^14^, ^15^ ^]^ Sillén phases,^[^ ^16^ ^]^ oxide perovskites,^[^ ^17^ ^]^ calcium, or sodium cobaltites^[^ ^18^, ^19^ ^]^) have been suggested as potential candidates to face such challenges. Currently, there are several p‐type oxide‐related compounds, namely cobaltites and oxyselenides, with zT values even above the threshold zT=1.^[^ ^14^, ^15^, ^20^ ^]^ On the contrary, the TE performance of n‐type oxides has so far been limited by their rather poor electrical transport properties. Among the n‐type oxides, ZnO is considered to be the most auspicious TE material.^[^ ^21^ ^]^ ZnO is a well‐known semiconductor with a simple hexagonal wurtzite crystal structure ($P6_{3}$ mc space group), high transmittance of visible light, large exciton binding energy, and a wide direct bandgap of ≈3.4 eV, which makes it promising for a broad range of optical and electronic applications.^[^ ^22^ ^]^ Indeed, ZnO‐based materials are already applied in piezoelectric nanogenerators, transparent thin‐film transistors, varistors, gas sensors, or solar cells.^[^ ^23^, ^24^, ^25^ ^]^ Despite a relatively high lattice thermal conductivity (≈5 W $m^{-1}$ $K^{-1}$ at 1273 K), an order of magnitude larger than for the layered p‐type oxides,^[^ ^26^, ^27^, ^28^ ^]^ ZnO is currently the best reported n‐type oxide with $zT_{\max}$ ≈ 0.4−0.44 for single‐doped ZnO at T>1000 K.^[^ ^27^, ^29^, ^30^, ^31^, ^32^ ^]^ Further enhancement of the TE performance, however, is hindered by pivotal obstacles such as the low solubility of dopant ions and novel enhancement principles are therefore required to effectively tune the electronic properties.

**Figure 1:**
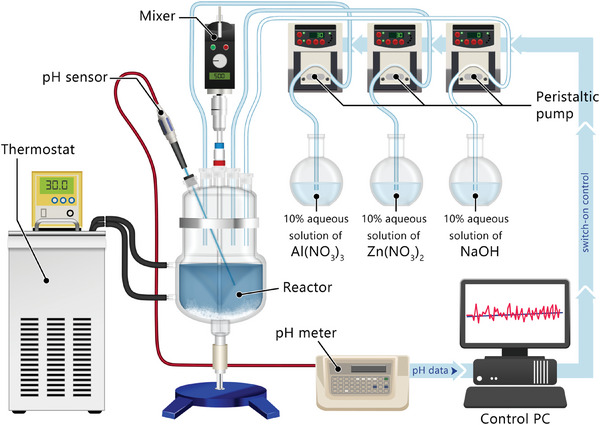
Schematic illustration of the semi‐automated laboratory‐made reactor used for chemical co‐precipitation.

Here, employing a sophisticated synthesis technique under strongly alkaline conditions (see **Figure** **1**), we experimentally realize a record‐high figure of merit for single‐doped ZnO, reaching zT=0.5 at 1100 K in ${\mathrm{Zn}}_{0.96}{\mathrm{Al}}_{0.04}O$. We find that this enhancement is related to intriguing changes in the electronic structure and Anderson‐like localization of charge carriers in the conduction band edge. In its essence, Anderson localization is the absence of diffusion of wave‐like objects (e.g., electrons in a solid) due to quantum interference of multiple self‐intersecting scattering paths.^[^ ^33^, ^34^, ^35^, ^36^ ^]^ In three dimensions, a transition occurs from a delocalized to a localized ground state when disorder surpasses a critical threshold with electrons becoming initially localized in the band tails.^[^ ^37^, ^38^ ^]^ Subsequently, when the Fermi level is near the band edge, distinctive temperature, and doping dependencies of charge transport emerge.^[^ ^39^, ^40^, ^41^ ^]^ Here, we provide evidence for such a scenario in our ZnO samples: i) variable range hopping conduction at low temperatures, ii) abnormally low charge carrier mobility, and iii) anomalous temperature and doping dependencies for the Seebeck coefficient and electrical conductivity. The bi‐dimensional optimization of charge carrier concentration and localization lead to a substantial enhancement of the thermoelectric power factor and figure of merit.

## 2 Results and Discussion

### 2.1 Microstructure Characterization

**Figure 2:**
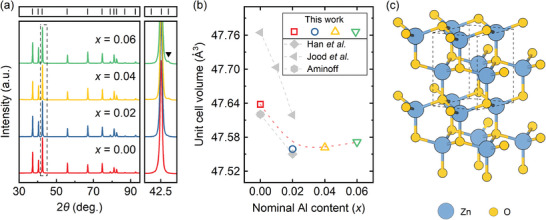
a) PXRD patterns of ${\mathrm{Zn}}_{1-x}{\mathrm{Al}}_{x}O$ (x = 0, 0.02, 0.04, 0.06) samples after SPS and annealing with an enlarged section (on the right) of a 2θ range from $42^{\circ }$ to $43^{\circ }$ where ${\mathrm{ZnAl}}_{2}O_{4}$ spinel phase has the most intensive reflection, which is indicated by a black solid triangle (▾). Bragg's reflections for the ZnO phase are indicated by black ticks on the top part of the figure. b) Calculated unit cell volumes for the ${\mathrm{Zn}}_{1-x}{\mathrm{Al}}_{x}O$ samples (empty symbols) compared to literature data (filled gray symbols).^[^ ^29^, ^42^, ^44^ ^]^ c) Illustration of the crystal structure of ZnO.

All ${\mathrm{Zn}}_{1-x}{\mathrm{Al}}_{x}O$ (*x* = 0, 0.02, 0.04, 0.06) samples had a relative density >97% after sintering and annealing consisting primarily of the ZnO phase with Wurtzite structure‐type and $P6_{3}mc$ space group (**Figures** **2**a,c).^[^ ^42^ ^]^ For samples with x>0.02, X‐ray diffraction (XRD) analyses and Rietveld refinements (Figure S1a–d, Supporting Information) reveal the formation of a ${\mathrm{ZnAl}}_{2}O_{4}$ spinel impurity phase with Gahnite structure‐type and the $Fd\bar{3}m$ space group;^[^ ^43^ ^]^ 1.2 and 2.2 vol.% for ${\mathrm{Zn}}_{0.96}{\mathrm{Al}}_{0.04}O$ and ${\mathrm{Zn}}_{0.94}{\mathrm{Al}}_{0.06}O$, respectively. No reaction residues, such as Zn(${\mathrm{NO}}_{3}$)_2_, Al(${\mathrm{NO}}_{3}$)_3_, or ${\mathrm{Al}}_{2}O_{3}$, were detected, confirming that the chemical co‐precipitation process was complete.

Substituting ${\mathrm{Zn}}^{2+}$ with ${\mathrm{Al}}^{3+}$ shrinks the unit cell due to the smaller ionic radius of the latter (0.6Å vs. 0.39Å in tetrahedral coordination, respectively^[^ ^45^ ^]^) as shown in Figure 2b. However, a noticeable reduction in the volume of the crystal unit cell was observed only for ${\mathrm{Zn}}_{0.98}{\mathrm{Al}}_{0.02}O$, while for samples with x>0.02, further reduction in volume did not occur (Figure 2b). This indicates that the solubility limit of Al in ZnO is reached between x=0.02 and 0.04, followed by the formation of ${\mathrm{ZnAl}}_{2}O_{4}$ spinel in the samples with x>0.02. We note that these findings consistently align with measurements of the charge carrier density (see Figure 6e), showing a pronounced increase as x goes from 0 to 0.02 and a slight additional increase for x=0.04. In addition, the introduction of Al into the ZnO lattice hinders the growth rate of the crystallites due to the impurity drag effect, leading to smaller crystallite sizes along with almost four times higher microstrain level (see Table S1, Supporting Information).

**Figure 3:**
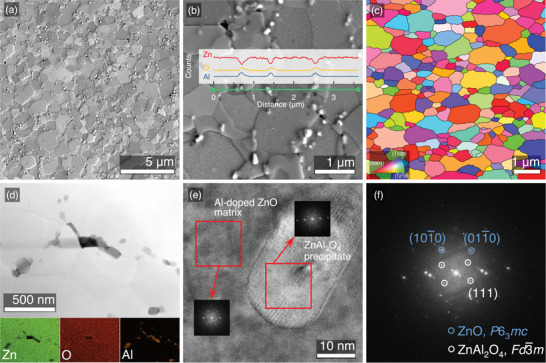
a) Mixed backscattered electron and secondary electron HRSEM micrograph, b) linear EDS analysis results obtained in HRSEM with low‐acceleration beam mode, c) EBSD mapping, d) combined STEM and energy‐dispersive spectroscopy (EDS) mapping images showing the spatial distribution of Zn, O, and Al elements (in green, red, and orange, respectively), e) high‐magnification transmission electron microscopy micrograph, revealing the lattice image of two crystalline phases, and f) region with crystal orientations, determined using Fourier transform spectra, for ${\mathrm{Zn}}_{0.96}{\mathrm{Al}}_{0.04}O$ sample.

**Figure 4:**
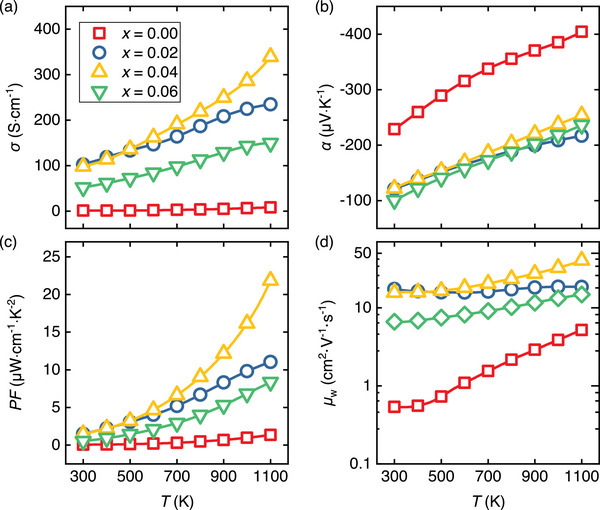
Temperature dependence of (a) electrical conductivity σ, b) Seebeck coefficient α, c) thermoelectric power factor $PF=\alpha^{2}\sigma$, and d) weighted mobility $\mu_{w}$ of ${\mathrm{Zn}}_{1-x}{\mathrm{Al}}_{x}O$ (x = 0, 0.02, 0.04, 0.06) prepared by chemical co‐precipitation.

**Figure 5:**
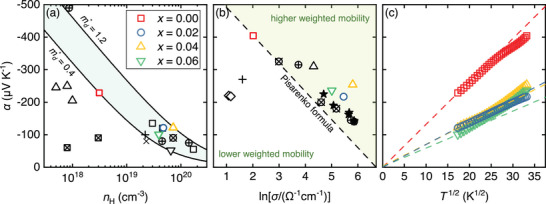
Seebeck coefficient of ${\mathrm{Zn}}_{1-x}{\mathrm{Al}}_{x}O$ (*x* = 0, 0.02, 0.04, 0.06) samples a) versus Hall carrier concentration (Pisarenko plot) at room temperature, b) versus logarithm of electrical conductivity (Jonker plot) at 1000 K, and c) versus temperature squared revealing variable range hopping conduction behavior $\alpha \left(T\right)\propto T^{1/2}$ below ≈600 K. The reference values are taken from: + ‐ Gayner et. al.,^[^ ^53^ ^]^ □ ‐ Berardan et. al.,^[^ ^54^ ^]^ ⊗ ‐ Tsubota et. al.,^[^ ^27^ ^]^ ▵ ‐ Jood et. al.,^[^ ^29^ ^]^ ⊕ ‐ Acharya et. al.,^[^ ^32^ ^]^ ★ ‐ Han et. al.,^[^ ^44^ ^]^ ∇ ‐ Nam et. al.,^[^ ^55^ ^]^ ◇ ‐ Mayandi et. al.,^[^ ^56^ ^]^ ⊠ ‐ Guan et. al.^[^ ^57^ ^]^

**Figure 6:**
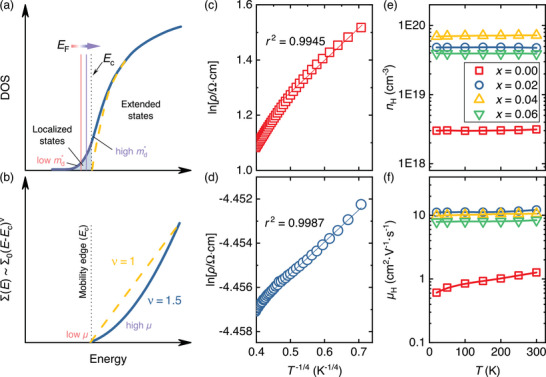
a) Sketch of the density of states (DOS) for a disordered material (solid line) with localized states in the band tails and an ordered material with parabolic band dispersion (dashed line). The localized states are separated from the delocalized (extended) states by a mobility edge $E_{c}$. When the Fermi level $E_{F}$ lies within the region of localized states, the system behaves insulator‐like, whereas metallic‐liked behavior is observed for $E_{F}>E_{c}$. b) Energy‐dependent transport distribution function near the parabolic band edge of a semiconductor with dominant acoustic phonon scattering (dashed line) and near the mobility edge of a disordered material showing a different energy dependence. Variable‐range‐hopping‐like dependence of ρ(T) at low temperatures for c) undoped ZnO and d) Al‐doped ${\mathrm{Zn}}_{0.98}{\mathrm{Al}}_{0.02}O$. e) Hall carrier concentration and f) Hall mobility of ${\mathrm{Zn}}_{1-x}{\mathrm{Al}}_{x}O$ (*x* = 0, 0.02, 0.04, 0.06). Anomalously low mobility is observed for undoped ZnO, which increases by an order of magnitude for ${\mathrm{Zn}}_{0.98}{\mathrm{Al}}_{0.02}O$, despite the simultaneous increase of the carrier concentration in agreement with a disorder‐modified DOS sketched in (a). Unconventional temperature dependencies of the Hall mobility, which increases with temperature, confirm this scenario.

In this section, we provide high‐resolution scanning electron microscopy (HRSEM) investigations to explore microstructural characteristics and elemental composition of the obtained ${\mathrm{Zn}}_{0.96}{\mathrm{Al}}_{0.04}O$ bulk, which is the sample demonstrating the highest TE performance (TE properties will be discussed in the next section). As shown in **Figure** **3**a, the pellet is densely packed with spinel precipitation distributed along the grain boundaries. In terms of grain size and texture, we performed an electron backscattered diffraction (EBSD) characterization presented in Figure 3c. We note that the sintered sample is isotropic and consists of shapeless sub‐micron‐sized grains with an average grain size of approximately 1 µm for ${\mathrm{Zn}}_{0.96}{\mathrm{Al}}_{0.04}O$ (the grain size distribution for other samples in this set is presented in Table S2, Supporting Information). Figure 3b demonstrates ${\mathrm{ZnAl}}_{2}O_{4}$ precipitates, primarily localized at the grain boundaries of the matrix phase, with an average size of 50nm (see Figure3b,d,e). To examine the composition of these precipitates, we conducted profile energy dispersive spectroscopy (EDS) analysis utilizing a low‐acceleration beam mode (3 kV) in the HRSEM to improve measurement precision. The resulting data are depicted in Figure 3b, revealing a significant increase in aluminum concentration within the precipitate, which is linked to the higher aluminum content in ${\mathrm{ZnAl}}_{2}O_{4}$ when compared to the Al‐doped ZnO matrix.

To characterize the microstructure of ${\mathrm{Zn}}_{0.96}{\mathrm{Al}}_{0.04}O$ at the nanoscale, EDS‐S/TEM imaging and scanning transmission electron microscopy (STEM) analyses were performed. STEM/EDS analysis was performed to map the sample on the nanoscale. Figure 3d displays the EDS mapping images, demonstrating the matrix with the spatial distribution of Zn, O, and Al. These results confirm that the formation of spinel precipitates occurs mainly along grain boundaries, presumably owing to heterogeneous nucleation. The high‐magnification transmission electron microscopy image (Figure 3e) confirms the interfacial boundary region between the Al‐doped ZnO matrix and ${\mathrm{ZnAl}}_{2}O_{4}$, which is also demonstrated by elemental mapping. The corresponding fast Fourier transformation (FFT) pattern shown in Figure 3f identifies two regions with different wurtzite ZnO and cubic ${\mathrm{ZnAl}}_{2}O_{4}$ phases.

### 2.2 Electrical Transport Properties

We studied the temperature‐dependent electrical conductivity σ and Seebeck coefficient α for all specimens in a broad temperature range of 300–1100 K. **Figure** **4**a–d display the temperature dependencies of σ, α, TE power factor $PF=\alpha^{2}\sigma$ and weighted mobility $\mu_{w}=\mu {\left(m^{*}/m_{e}\right)}^{3/2}$ for ${\mathrm{Zn}}_{1-x}{\mathrm{Al}}_{x}O$ with x = 0, 0.02, 0.04, and 0.06. The electrical conductivity increases by two orders of magnitude when Al is incorporated (Figure 4a) due to an increase of the charge carrier concentration from the aliovalent substitution of ${\mathrm{Zn}}^{2+}$ with ${\mathrm{Al}}^{3+}$:

(1)

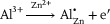




At this point, we note that our investigations of $\mu_{w}$ and the Hall mobility $\mu_{H}$ also reveal an unexpected simultaneous enhancement of the carrier mobility upon Al doping by more than one order of magnitude (see Figure S8, Supporting Information), which will be discussed later. Both the Hall carrier concentration $n_{H}$ (Figure 6e) and the electrical conductivity σ (Figure 4a) increase with Al doping up to x=0.04. The higher carrier concentration and the presence of spinel phase inclusions comprising 1.2 vol.% in the ${\mathrm{Zn}}_{0.96}{\mathrm{Al}}_{0.04}O$ sample confirm that the solubility limit of Al in ${\mathrm{Zn}}_{1-x}{\mathrm{Al}}_{x}O$ in this study lies between x=0.02−0.04. Further increase of the spinel phase resulted in a reduction of both $n_{H}$ and $\mu_{H}$, leading to a decrease of σ for ${\mathrm{Zn}}_{0.94}{\mathrm{Al}}_{0.06}O$. The sharp increase in electrical conductivity observed in Al‐doped ${\mathrm{Zn}}_{1-x}{\mathrm{Al}}_{x}O$ at high temperatures is associated with the excitation of carriers from Al donor levels situated below the conduction band edge; thermally activated electrical conductivity data are shown in Figure S9 (Supporting Information).

Due to intrinsic defects being present even in pristine ZnO, such as oxygen vacancies, zinc interstitials or hydrogen substitution observed in samples obtained via wet chemistry methods,^[^ ^46^, ^47^, ^48^ ^]^ the Fermi level is pinned close to the conduction band edge, resulting in n‐type behavior. As the chemical potential is shifted deeper into the conduction band upon Al doping (all details, including chemical potential values, etc., are shown in Table S3, Supporting Information), the Seebeck coefficient is obviously decreased in absolute values. However, we stress that the Seebeck effect in our Al‐doped samples remains extraordinarily high (up to −250 $\mu V\;K^{-1}$ around 1100 K), compared to previous reports on single‐doped ${\mathrm{Zn}}_{1-x}{\mathrm{Al}}_{x}O$ (|α|≈ 130−170 $\mu V\;K^{-1}$) despite comparable carrier concentrations, which we trace back to the partial localization and suppression of charge carriers in the conduction band edge as discussed in the following section.

In Figure 4c, we compare the power factor ($PF=\alpha^{2}\sigma$) of our samples, which increases monotonically and reaches a maximum $PF_{\max}=21.5\;\mu W\;{\text{cm}}^{-1}\;K^{-2}$ at 1100 K for ${\mathrm{Zn}}_{0.96}{\mathrm{Al}}_{0.04}O$, constituting one of the highest ever reported values for single‐doped ZnO ceramics.^[^ ^27^, ^28^, ^29^, ^44^ ^]^ By utilizing the experimental electrical conductivity and Seebeck coefficient, we also calculated the weighted mobility $\mu_{w}$,^[^ ^49^ ^]^ depicted in Figure 4d. For undoped ZnO, we find exceptionally small values of $\mu_{w}$, about two orders of magnitude smaller than those reported for other high‐performance TE materials.^[^ ^49^, ^50^ ^]^ Moreover, $\mu_{w}$ increases both with temperature and doping, hinting at unconventional and anomalous carrier scattering in ${\mathrm{Zn}}_{1-x}{\mathrm{Al}}_{x}O$. To further analyze the electronic transport in our samples, we studied the Seebeck coefficient as a function of the carrier concentration, which can be described by the Pisarenko relation:^[^ ^51^ ^]^

(2) $$\begin{array}{c} \alpha =\pm \frac{k_{B}}{e}\left[\frac{5}{2}+r+\ln\frac{2{\left(2\pi m_{d}^{*}\;k_{B}\;T\right)}^{3/2}}{h^{3}n}\right] \end{array}$$

Here, $k_{B}$ denotes the Boltzmann constant, r the scattering factor (r=−1/2 for acoustic phonon scattering), $m_{d}^{*}$ the density‐of‐states effective mass and h Planck's constant. **Figure** **5**a shows a Pisarenko plot for Al‐doped ZnO samples from the literature as well as those studied in the present work. It can be clearly seen that while undoped ZnO exhibits a density‐of‐states effective mass ≈0.4 times the free electron mass $m_{e}$, $m_{d}^{*}$ increases by almost a factor three upon Al substitution ($m_{d}^{*}\approx 1.2\;m_{e}$). This is at odds with the simple notion of a single conduction band with parabolic band dispersion, as predicted from density functional theory (DFT) calculations.^[^ ^52^ ^]^ Simultaneously, the weighted mobility of charge carriers increases with Al doping up to x=0.04 in ${\mathrm{Zn}}_{1-x}{\mathrm{Al}}_{x}O$ (see Figure 5b). Again this contradicts the above‐mentioned scenario of a single parabolic band (SPB) with dominant acoustic phonon scattering.

Instead, as will be elucidated below, we find that all of the observed transport properties, i.e., the temperature‐dependent electrical conductivity, Seebeck coefficient, Hall carrier concentration, and Hall mobility, can be consistently and adequately described within the framework of Anderson localization of charge carriers in the band edge states caused by intrinsic defects and disorder in our ZnO‐based samples. Indeed, a closer look at the temperature‐dependent Seebeck coefficient α(T) already hints at a peculiar charge transport mechanism where conduction takes place via hopping between localized band edge states that are close in energy space, called variable range hopping (VRH). This leads to a characteristic temperature dependence of the Seebeck coefficient $\alpha \left(T\right)\propto T^{1/2}$ for 3D systems.^[^ ^58^ ^]^ Figure 5c shows that α(T) is consistent with VRH in undoped ZnO and Al‐doped ${\mathrm{Zn}}_{0.98}{\mathrm{Al}}_{0.02}O$ at least up to T=600K as the data linearly extrapolate to α=0 at T→0 when plotting α versus $T^{1/2}$. Deviations occur at higher temperatures when the conduction of electrons becomes a combination of VRH, thermal activation across the mobility edge, and diffusive transport of electrons moving more or less freely within the extended states resulting in a complex interplay of different transport mechanisms. Yamamoto et al. recently proposed a modified theoretical scheme, based on the Sommerfeld‐Bethe relation, for the low‐temperature Seebeck coefficient in the case of VRH conduction, yielding $\alpha \left(T\right)\propto T^{d/(d+1)}$ for a d dimensional system.^[^ ^59^ ^]^ However, we note that $\alpha \left(T\right)\propto T^{3/4}$ describes our data with less accuracy than the conventional $\alpha \left(T\right)\propto T^{1/2}$.

### 2.3 Anderson Localization in ZnO

In materials with significant disorder, such as our samples, the band edge changes from a parabolic (DOS $\propto E^{1/2}$) to a Gaussian shape (see **Figures** **6**a,b). Moreover, the multiple carrier scattering off of strong random potential fluctuations can lead to partial localization of electronic states near the band edge and below a critical energy level referred to as a mobility edge $E_{c}$.^[^ ^37^ ^]^ Specifically, when the Fermi level $E_{F}$ falls below the mobility edge, the behavior of carrier transport becomes activated, i.e., phonon‐assisted hopping conduction from one localized state to another or thermal excitation into the delocalized states across the mobility edge. Conversely, if $E_{F}$ lies above $E_{c}$ within the extended states, the material exhibits conventional metallic behavior. The energy‐dependent transport distribution function (see Figure 6b) increases with $\Sigma \left(E\right)\propto \Sigma_{0}{\left(E-E_{c}\right)}^{\nu }$ at the mobility edge, where ν≈1.5 is the critical exponent of the so‐called Anderson transition,^[^ ^60^ ^]^ which occurs when the chemical potential passes through the mobility edge either from the insulating toward the metallic side or vice versa. As demonstrated recently, such modification of the energy‐dependent transport function in the presence of disorder can shift the optimal doping level for maximum zT and, more importantly, further become the source of enhanced TE performance.^[^ ^61^, ^62^, ^63^, ^64^ ^]^

Looking back at Figure 4 in detail, it becomes evident that the transport properties of undoped and Al‐doped ZnO cannot be adequately explained assuming a single parabolic band with a well‐defined value of the effective mass, as well as dominant acoustic phonon scattering. While the Seebeck coefficient's absolute values monotonically increase over the measured temperature range, as expected for semiconductors where the chemical potential resides deep within the conduction or valence band, the electrical conductivity exhibits a semiconductor‐like increase with temperature dσ/dT>0. This behavior is typically associated with a scenario where the chemical potential is inside or close to the energy gap. Another intriguing feature is the very weak temperature dependence of the electrical conductivity. To gain deeper insights into the transport properties, we conducted investigations on the temperature‐dependent electrical resistivity and Hall effect at low temperatures ranging from 4 to 300 K.

In Figure 6c,d, we show ρ(T) for undoped ZnO and ${\mathrm{Zn}}_{0.98}{\mathrm{Al}}_{0.02}O$ samples, respectively (normal resistivity curves ρ vs T plotted in Figure S6, Supporting Information). Both samples show a more or less linear dependence of ρ(T) in rectifying coordinates (lnρ vs $T^{-1/4}$) at low temperatures, hinting at the possibility of a 3D variable range hopping mechanism (VRH). While the curves are not perfectly linear, deviations may be expected when the Fermi energy is near the mobility edge because charge carriers can get excited into the extended states through nearest‐neighbor hopping and charge carriers within the extended states can also contribute in a diffusive manner. This can make the overall T‐dependent behavior more complicated. It is crucial to emphasize that alternative hopping mechanisms, including nearest‐neighbor, Efros‐Shlovskii, and also weak localization, provide a far less accurate description of our experimental data. In Figure 6e,f, we present the Hall carrier concentration $n_{H}$ and Hall mobility $\mu_{H}$, respectively, extracted from the Hall coefficient assuming a one‐band model. Remarkably, both undoped ZnO, and Al‐doped ZnO exhibit nearly no variation in the carrier concentration with temperature. This finding suggests that the semiconductor‐like behavior of the electrical conductivity (dσ/dT>0) is not caused by an increase in the charge carrier concentration. Instead, it results from an anomalous trend in the Hall mobility, which very slightly increases with temperature, contradicting the idea of charge carrier transport being dominated by electron–phonon scattering. Similar observations have been made for other systems with a mobility edge close to the Fermi level such as ${\mathrm{In}}_{2}O_{3-x}$,^[^ ^65^ ^]^ ${\mathrm{Bi}}_{2+x}{\mathrm{Sr}}_{2-x}{\mathrm{CuO}}_{y}$,^[^ ^66^ ^]^ $(\mathrm{GeTe})x({\mathrm{Sb}}_{2}{\mathrm{Te}}_{3})100-x$ and ${\mathrm{GeSb}}_{2}{\mathrm{Te}}_{4}$ films with varying degree of structural disorder^[^ ^61^ ^]^ as well as the full‐Heusler compound ${\mathrm{Fe}}_{2}\mathrm{VAl}$ with intrinsic antisite defects.^[^ ^62^ ^]^ If $E_{F}$ is situated just below the mobility edge (as we expect for our ZnO‐based materials) more and more electronic states above $E_{c}$ contribute to transport as the Fermi‐Dirac distribution broadens with temperature, consequently increasing the carrier mobility. Another indication that this is a plausible scenario is given by the fact that $\mu_{H}$ increases also when doping Al on the Zn site. Typically, $\mu_{H}$ decreases when $n_{H}$ increases in semiconductors. However, while the charge carrier concentration increases from $3.1\cdot 10^{18}$ cm^−3^ in undoped ZnO to $4.8\cdot 10^{19}$ cm^−3^ in ${\mathrm{Zn}}_{0.98}{\mathrm{Al}}_{0.02}O$, $\mu_{H}$ is simultaneously enhanced by one order of magnitude as well. We recall that an almost identical behavior was found for the weighted mobility, extracted from the Seebeck coefficient and electrical conductivity (see Figure 4d). This is consistent with a picture where $E_{F}$ is shifted closer to $E_{c}$ and therefore closer toward the delocalized (extended) states. A disorder‐modified DOS, as depicted in Figure 6a, also explains the seemingly odd variation of the SPB parameters with Al doping, such as $m_{d}$ and $\mu_{w}$ in Figures 5a, b. Since the band edge changes to a Gaussian‐like shape, an increase of $m_{d}$ is reasonable and well explained when the Fermi energy is shifted further into the band. Moreover, whereas a simple SPB model would predict a decrease of $\mu_{w}$ when $m_{d}$ increases, the simultaneous enhancement of these SPB parameters follows naturally from a picture of Anderson‐localized band edge states.

In summary, transport properties of both undoped and Al‐doped ZnO can be consistently explained within the framework of Anderson localization of electronic states inside the band edges and a mobility edge close to the Fermi level, in agreement with earlier reports on this system.^[^ ^67^, ^68^, ^69^ ^]^ The suppression of charge carriers with energies close to the mobility edge leads to a modification of the energy‐dependent transport distribution function Σ(E), boosting the TE performance.^[^ ^61^, ^62^ ^]^

### 2.4 Thermal Transport Properties

**Figure 7:**
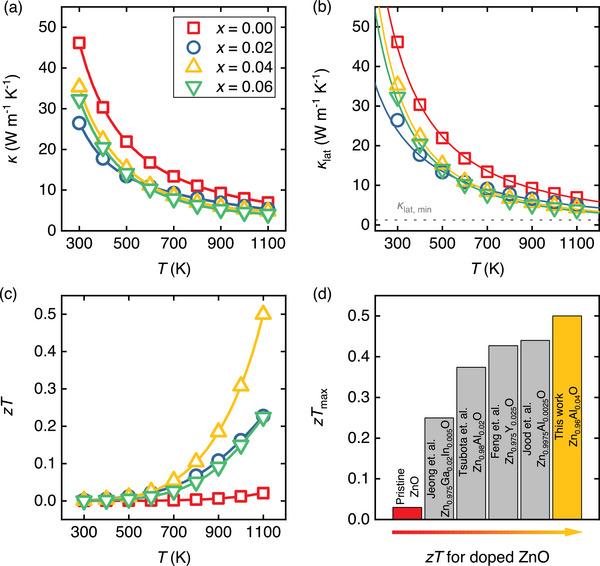
Temperature dependence of (a) total and (b) lattice thermal conductivity, and (c) dimensionless figure of merit (zT) of ${\mathrm{Zn}}_{1-x}{\mathrm{Al}}_{x}O$ (x = 0, 0.02, 0.04, 0.06) samples. In (b) solid lines are calculated by the Debye–Callaway model modified by Glassbrenner and Slack for high‐temperature regions, where $T\gg \theta_{D}$.^[^ ^70^ ^]^ Dashed line in (b) is the minimum lattice thermal conductivity (glass limit) calculated from Cahill's model.^[^ ^71^ ^]^ d) Comparison of maximum zT achieved for doped ZnO‐based ceramics.

The temperature‐dependent thermal conductivity κ(T) of ${\mathrm{Zn}}_{1-x}{\mathrm{Al}}_{x}O$ is displayed in **Figure** **7**a. The incorporation of Al atoms on the Zn sublattice reduces κ across the entire measured temperature range. We note that a small substitution of only 2 at.% of Al for Zn leads to a twofold reduction in room‐temperature thermal conductivity. This reduction is associated with two important factors: i) a decrease in the average grain size by one order of magnitude and ii) the presence of ${\mathrm{Al}}_{\text{Zn}}$ point defects. For ${\mathrm{Zn}}_{1-x}{\mathrm{Al}}_{x}O$ with x=0.04 and x=0.06, we observe a substantial increase in room‐temperature thermal conductivity compared to the sample with x=0.02. This increase is attributed to the formation of ${\mathrm{ZnAl}}_{2}O_{4}$ precipitates with relatively high thermal conductivity.

To further understand the thermal transport behavior, we analyzed the lattice component of thermal conductivity $\kappa_{\text{lat}}$ (see Figure 7b) using the Wiedemann‐Franz law $\kappa_{\text{lat}}=\kappa -L\sigma T$, where L is the Lorenz number, which has been obtained from an estimate using experimental values of the Seebeck coefficient α,^[^ ^72^ ^]^ widely used for TE materials.^[^ ^73^, ^74^ ^]^ We find that Umklapp‐processes are the dominant phonon scattering mechanism as the lattice thermal conductivity follows a $T^{-1}$ behavior for undoped ZnO and ${\mathrm{Zn}}_{0.98}{\mathrm{Al}}_{0.02}O$. However, for samples beyond the Al solubility limit in which we detect the spinel phase, a more intricate and complex interplay of phonon scattering mechanisms emerges, resulting in a stronger temperature dependence of $\kappa_{\text{lat}}\propto T^{-1.5}$. The experimental data of $\kappa_{\text{lat}}$ were fitted and analyzed by the Debye‐Callaway model modified by Glassbrenner and Slack for high temperatures above the Debye temperature ($T\gg \theta_{D}$) since the bare Debye‐Callaway model is insufficient at predicting the lattice thermal conductivity (more details are presented in the Supporting Information section). In the model by Glassbrenner and Slack, the lattice thermal conductivity is given by

(3) $$\begin{array}{c} \kappa_{\text{lat}}=\frac{k_{B}}{2\pi^{2}\upsilon_{a}}{\left(\frac{k_{B}T}{\hbar }\right)}^{3}\int_{0}^{\theta_{D}/T}\tau_{c}x^{2}dx \end{array}$$

where $x=\hbar \omega /k_{B}T$, $\upsilon_{a}$ is the average velocity, and $\tau_{c}$ is the total phonon relaxation time. We accounted for point defects and Umklapp scattering. Combining these scattering mechanisms via Mathiessen's rule (see Supporting Information for additional details), the calculated lattice thermal conductivities are shown in Figure 7b as solid lines, together with the minimal theoretical $\kappa_{\text{lat,min}}=1.22$ $W\;m^{-1}\;K^{-1}$ in the ZnO system calculated by Cahill's model (dashed line).^[^ ^71^ ^]^ As expected from the mass difference between Zn and Al (65.38  and 26.98 $g\;{\text{mol}}^{-1}$, respectively) along with the size difference (0.6  and 0.39 Å, respectively),^[^ ^45^ ^]^ the introduction of Al increases the mass and strain field fluctuations. Thus, point defect scattering plays the main role in decreasing the thermal conductivity. Furthermore, fitting reveals that Al doping leads to an increase in the disorder scattering parameter and strain field fluctuation (see Table S5, Supporting Information), resulting in $\kappa_{\text{lat}}=3.9\;W\;m^{-1}\;K^{-1}$ at 1100 K for ${\mathrm{Zn}}_{0.94}{\mathrm{Al}}_{0.06}O$.

### 2.5 Thermoelectric Figure of Merit

Figure 7c shows the dimensionless figure of merit zT of our ${\mathrm{Zn}}_{1-x}{\mathrm{Al}}_{x}O$ samples as a function of temperature, as well as a comparison of the maximum zT obtained at 1100 K in this study and previous achievements in doped ZnO^[^ ^27^, ^28^, ^29^, ^30^, ^31^, ^32^ ^]^ (Figure 7d). In the present study, we achieved a sizeable zT=0.5 at 1100 K for ${\mathrm{Zn}}_{0.96}{\mathrm{Al}}_{0.04}O$, constituting one of the highest values achieved thus far in bulk ZnO ceramics. We identify the substantial improvement in zT as a synergistic result of Anderson‐like localization of charge carriers in the band edge and a unique microstructure obtained by a combination of the chemical co‐precipitation and SPS technique. The phenomenon of Anderson localization, however, which affects the confinement of charge carriers in the band edge states, is not limited to the materials investigated in our study. Instead, we stress that it should be regarded as a fundamental principle with broader applicability to other disordered materials.

## 3 Conclusion

In summary, we strongly enhance the TE performance of Al‐doped ${\mathrm{Zn}}_{1-x}{\mathrm{Al}}_{x}O$, synthesized via chemical co‐precipitation and consecutive spark plasma sintering, which allows one to tailor and fine‐tune the microstructure and defects in ZnO‐based materials. Intrinsic defects and structural disorder alter the electronic structure, causing Anderson‐like localization of charge carriers in the band edge, manifested in characteristic temperature and doping dependencies of all electronic transport properties. The modification of energy‐dependent transport close to the Fermi level shifts the optimal carrier concentration in Al‐doped ZnO and consequently leads to exceptional values of the TE power factor up to 21.5 µW cm^−1^ K^−2^, resulting in zT=0.5 at 1100 K, demonstrating the benefit of utilizing disorder‐induced charge localization as an additional tuning parameter. Our work presents a novel concept for achieving high TE performance in ZnO‐based systems that should be applicable to many other TE materials inherently prone to defects and disorder.

## 4 Experimental Section

### Synthesis

Chemically pure zinc nitrate $\mathrm{Zn}{\left({\mathrm{NO}}_{3}\right)}_{2}\cdot 6H_{2}O$, aluminum nitrate $\mathrm{Al}{\left({\mathrm{NO}}_{3}\right)}_{3}\cdot 9H_{2}O$, and sodium hydroxide NaOH were used as raw materials and reducing agent, respectively. The precipitation experiments were carried out at room temperature using a semi‐automated laboratory‐made reactor shown in Figure 1. ${\mathrm{Zn}}_{1-x}{\mathrm{Al}}_{x}O$ (x = 0, 0.02, 0.04, 0.06) powders were prepared by chemical co‐precipitation directly from the 10% aqueous solution of both Al and Zn nitrates in deionized water (Milli‐Q Water, Millipore, Germany) in required ratios. The precipitation had been performed by titrating both 10% aqueous solutions of nitrates and NaOH into the water under continuous stirring (see Figure 1). The pH value was kept around 13 by adjusting the titration rate of NaOH solution into the reactor. Upon completion of the process, the resulting solution was subjected to decantation until the pH value reached 7. The as‐prepared gel was centrifuged at 4000 rpm for 10 min (Rotanta 460, Hettich, Germany) and washed with distilled water. This procedure was repeated three times. Finally, the white powder was collected and dried on filter paper at room temperature overnight. The powder was further calcined at 973 K for 1 h in air. After the heat treatment, the obtained powder was ball milled using a Pulverisette 7 planetary micro mill (Fritsch, Germany) with corundum balls (18 balls, Ø = 10 mm) and zirconium dioxide jars (45 ml) in the air at 400 rpm for 15 min using a powder‐to‐ball mass ratio of 1:5. The obtained powder was sintered in a Ø15 mm graphite die under an axial compressive stress of 50 MPa at 1273 K in vacuum (≈3 Pa) using a Dr.Sinter‐1080 spark plasma sintering (SPS) system (Fuji‐SPS, Japan). The heating speed was 100 K min^−1^, and the dwelling time was 8 min followed by free cooling down to room temperature. All the fabricated bulks were annealed at 1273 K for 10 h in an air atmosphere.

### Compositional and Structural Analysis

Powder X‐ray diffraction (PXRD) patterns were collected at room temperature using a DRON‐4 diffractometer (IC Bourevestnik, Russia) equipped with a CoKα (λ = 1.7903 Å) X‐ray tube. Rietveld refinement was performed using the self‐developed software package.^[^ ^75^ ^]^ The microstructure and elemental composition of the sintered samples were examined by scanning electron microscopy and energy‐dispersive X‐ray spectroscopy (EDS) on ultra‐high resolution HRSEM SU8230 (Hitachi, Japan) in conjunction with an X‐${\mathrm{Max}}^{N}$ Horiba EDS detector (Horiba, Japan) and Symmetry S3 EBSD detector (Oxford Instruments, UK). Electron Backscatter Diffraction (EBSD) data were analyzed using the open‐source MTEX software package. Lattice images were acquired using a JEM‐3100FEF (JEOL, Japan) transmission electron microscope (TEM), operating at an acceleration voltage of 300 kV. The high‐angle annular dark‐field scanning transmission electron microscopy (HAADF‐STEM) image was taken with a condenser lens aperture of 70 µm, a beam‐convergence semi‐angle of approximately 6.8 mrad, and a camera length of 100 mm. The elemental mapping data were obtained using a JED‐2300 (JEOL, Japan) series EDS equipment, and the EDS acquisition time per pixel was fixed at 0.2 ms.

### Transport Measurements

Temperature dependencies of the electrical conductivity σ and the Seebeck coefficient α were simultaneously measured along the radial direction of bar‐shaped samples with dimensions of 10 mm × 3 mm × 1.5 mm by the standard 4‐probe and differential methods, respectively. The measurements were conducted under a He atmosphere using a custom‐made system (Cryotel, Russia). Thermal conductivity κ was determined from thermal diffusivity measured in the axial direction of a square‐shaped sample with dimensions of 10 mm × 1.5 mm. The thermal conductivity was calculated as $\kappa =\chi \cdot C_{p}\cdot d$, where χ is the thermal diffusivity, $C_{p}$ is the specific heat capacity estimated by the Debye model,^[^ ^76^ ^]^ and d is the bulk density determined using the Archimedes method. Temperature dependence of thermal diffusivity was measured by the laser flash diffusivity method (LFA 457 MicroFlash, Netzsch, Germany). To minimize errors from the emissivity and translucency of the material to infrared radiation, the samples were usually covered with a thin layer of graphite. However, at high temperatures, zinc oxide reacts with carbon forming elemental zinc and carbon monoxide. Thus, the specimens were pre‐coated with a ≈100 nm thickness layer of platinum in order to prevent the reaction between ZnO and graphite. The thermal diffusivity data were analyzed using a Cape‐Lehman model with pulse correction.^[^ ^77^ ^]^ The temperature dependence of the Hall constant was measured using the stepwise rotation technique with a laboratory‐made system (Cryotel, Russia). The Hall constant $R_{H}$ was calculated from the angular dependence of the Hall resistivity $\rho_{H}\left(\phi \right)$ under a fixed magnetic field of H= 3 T applied perpendicular to the rotation axis. The Hall signal modulation by a simple cosine law $\rho_{H}\left(\phi \right)=\rho_{H_{0}}+\rho_{H_{1}}cos\phi$ allowed the calculation of the harmonic component $\rho_{H_{1}}$, which was used to determine the Hall coefficient as $R_{H}=\rho_{H_{1}}/H$. The Hall carrier concentration $n_{H}$, and Hall carrier mobility $\mu_{H}$ were calculated as $n_{H}=1/\left(e\cdot R_{H}\right)$ and $\mu_{H}=\sigma \cdot R_{H}$, respectively. The Hall measurement accuracy was within 5–10%, while the uncertainty in the measured transport properties was estimated to be 6% for the Seebeck coefficient, 8% for the electrical conductivity, 11% for the thermal conductivity, and 19% for the figure of merit zT.^[^ ^78^ ^]^ To improve the readability of the figures, the error bars are not shown.

## Conflict of Interest

The authors declare no conflict of interest.

## Supporting information

Supporting Information

## Data Availability

The data that support the findings of this study are available from the corresponding author upon reasonable request.
