# Supplementary material for: Record‐High Thermoelectric Performance in Al‐Doped ZnO via Anderson Localization of Band Edge States
Source: Adv Sci (Weinh). 2024 May 5;11(26):2309291. doi: 10.1002/advs.202309291 (PMC11234415; doi:10.1002/advs.202309291)
Supplement: Supplementary file 1 — Supporting Information [file ADVS-11-2309291-s001.pdf]

ADVANCED SCIENCE
Open Access

Supporting Information

for *Adv. Sci.*, DOI 10.1002/advs.202309291

Record-High Thermoelectric Performance in Al-Doped ZnO via Anderson Localization of Band Edge States

*Illia Serhiienko, Andrei Novitskii, Fabian Garmroudi, Evgeny Kolesnikov, Evgenia Chernyshova, Tatyana Sviridova, Aleksei Bogach, Andrei Voronin, Hieu Duy Nguyen, Naoyuki Kawamoto, Ernst Bauer, Vladimir Khovaylo and Takao Mori**

— Supporting Information —

Record-High Thermoelectric Performance in Al-Doped ZnO via Anderson Localization of Band Edge States

Illia Serhiienko,[†,‡] Andrei Novitskii,[†] Fabian Garmroudi,[¶] Evgeny Kolesnikov,[§] Evgenia Chernyshova,[§] Tatyana Sviridova,[§] Aleksei Bogach,[‖] Andrei Voronin,[§] Hieu Duy Nguyen,[⊥] Naoyuki Kawamoto,[⊥] Ernst Bauer,[¶] Vladimir Khovaylo,[#,§] and Takao Mori[*,†,‡]

†*International Center for Materials Nanoarchitectonics (WPI-MANA), National Institute for Materials Science (NIMS), Tsukuba, Ibaraki 305-0044, Japan.*

‡*Graduate School of Pure and Applied Sciences, University of Tsukuba, Tsukuba, Ibaraki 305-8573, Japan.*

¶*Institute of Solid State Physics, TU Wien, Vienna A-1040, Austria.*

§*National University of Science and Technology MISIS, Moscow 119049, Russia.*

‖*Prokhorov General Physics Institute of the Russian Academy of Sciences, Moscow 119991, Russia.*

⊥*Center for Basic Research on Materials (CBRM), National Institute for Materials Science (NIMS), Tsukuba, Ibaraki 305-0044, Japan.*

#*Belgorod State University, Belgorod 308015, Russia.*

E-mail: MORI.Takao@nims.go.jp

Rietveld refinement for powder X-ray diffraction patterns

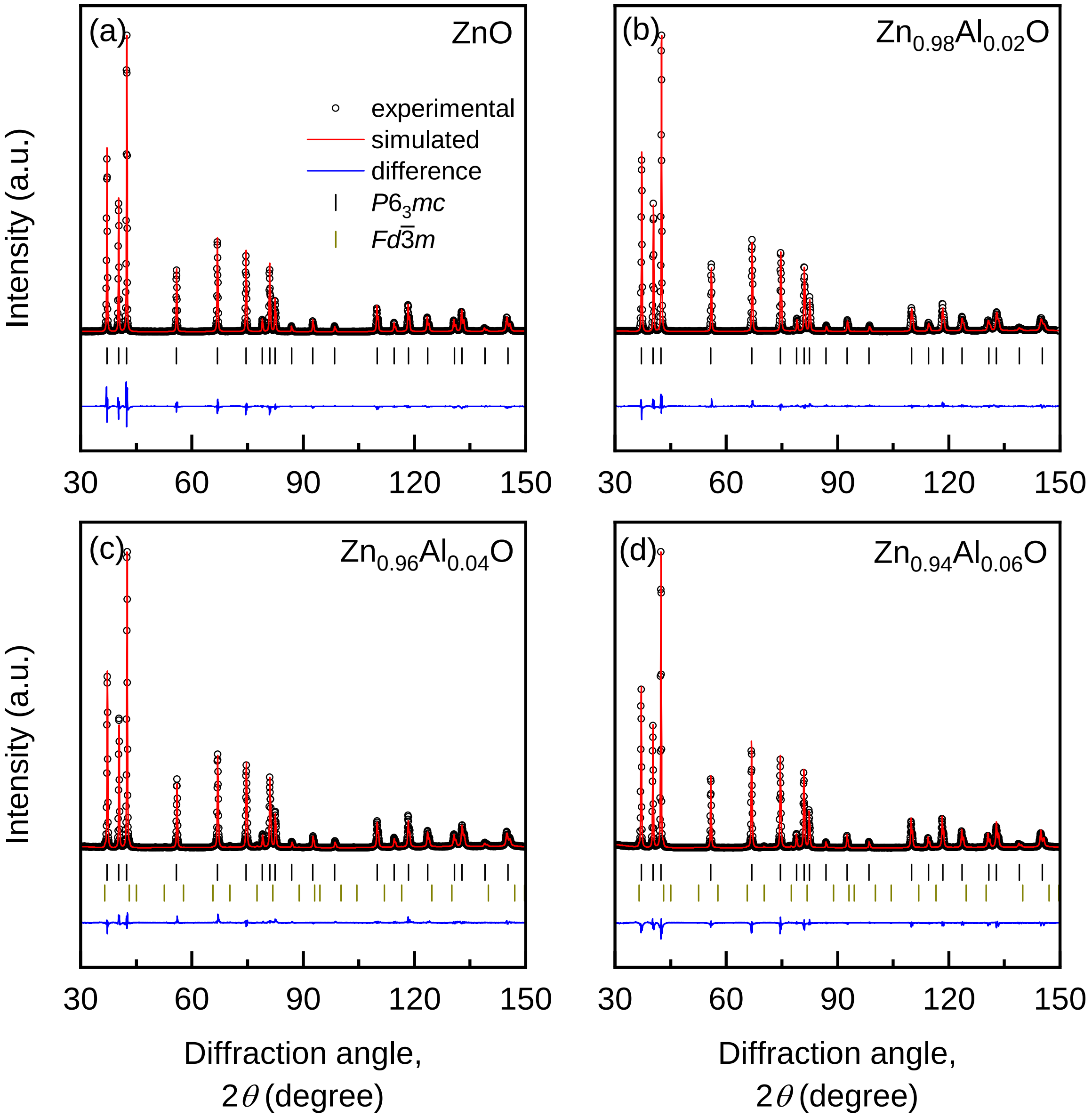

Figure S1: Rietveld refinement for the $Zn_{1-x}Al_xO$ (x = 0, 0.02, 0.04, 0.06) samples.

Table S1: Nominal Al content x, unit cell volume V_{cell}, crystalline size calculated in different directions D_{hkl}, microstrain ε, $ZnAl_2O_4$ volume fraction ϕ, weighted profile R-factor R_{wp}, intensity R-factor R_p, and goodness of fit, GoF of the $Zn_{1-x}Al_xO$ (x = 0, 0.02, 0.04, 0.06) samples.

Nominal composition	V_{cell} (Å^3)	D_{100} (nm)	D_{001} (nm)	ε (%)	ϕ (%)	R_{wp} (%)	R_p (%)	GoF
ZnO	47.6377	253	254	0.03	0	8.9	7.1	1.73
$Zn_{0.98}Al_{0.02}O$	47.5589	150	130	0.11	0	7.1	5.5	1.39
$Zn_{0.96}Al_{0.04}O$	47.5616	140	120	0.10	1.2	8.2	6.1	1.75
$Zn_{0.94}Al_{0.06}O$	47.5713	141	123	0.10	2.3	7.0	5.2	1.55

Field emission scanning electron microscopy (SEM) and electron backscatter diffraction (EBSD) analysis

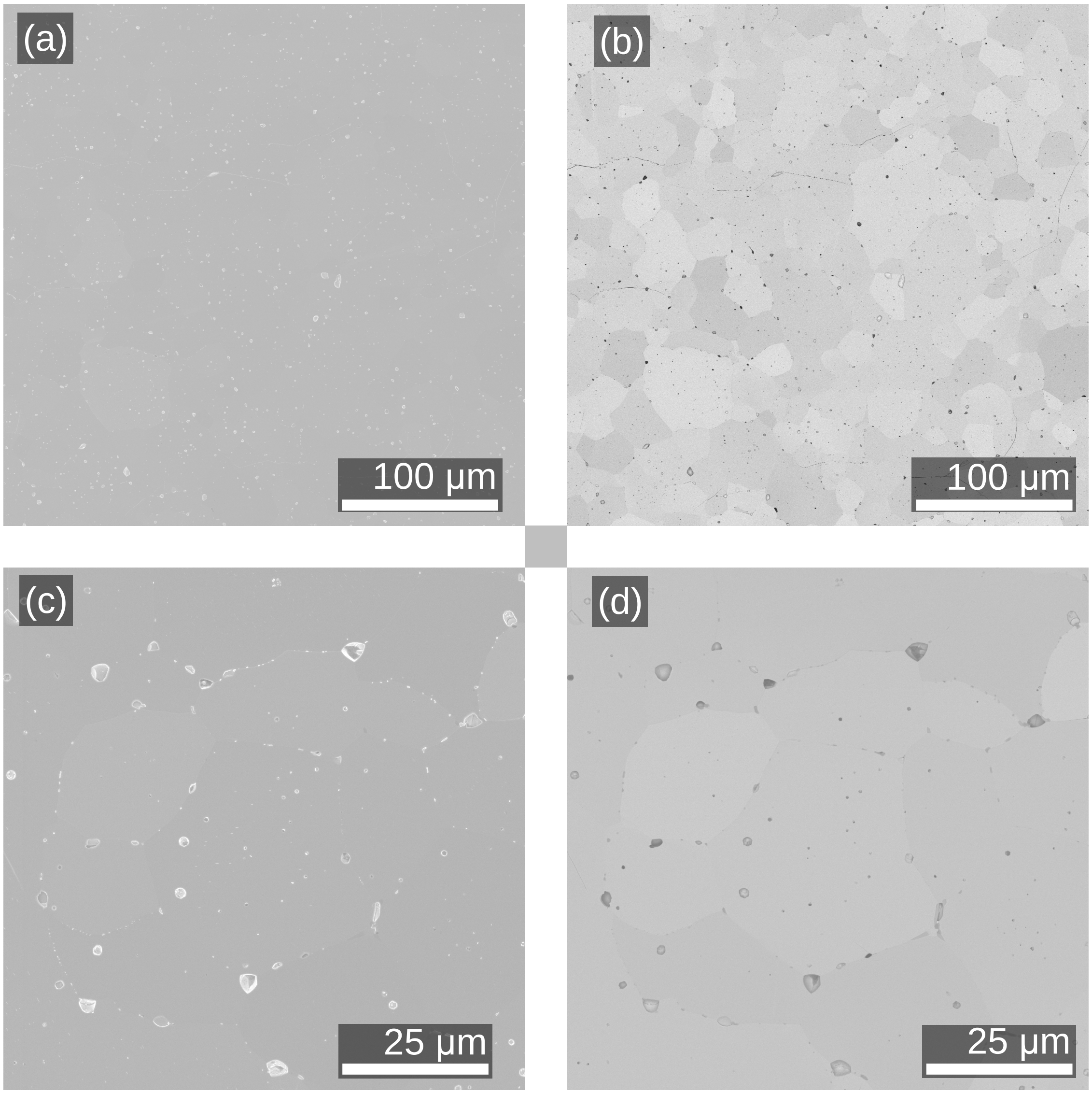

Figure S2: FE-SEM microphotographs of undoped ZnO polished surface in (a,c) backscattered electron mode, and (b,d) mixed backscattered and secondary electron mode.

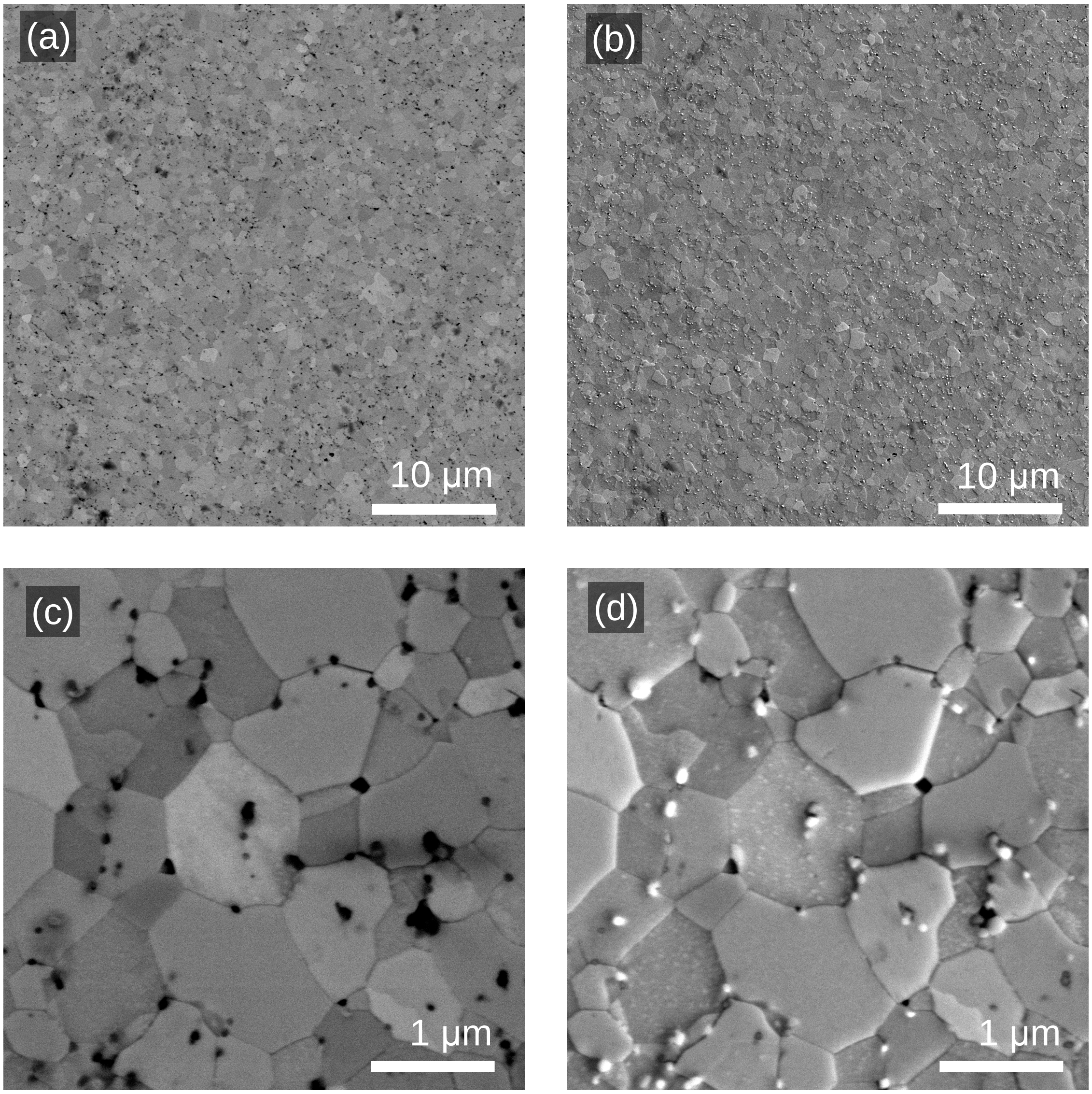

Figure S3: FE-SEM microphotographs of $Zn_{0.96}Al_{0.04}O$ polished surface in (a,c) backscattered electron mode, and (b,d) mixed backscattered and secondary electron mode.

Table S2: Average grain size estimated from SEM images D_{SEM} of the $Zn_{1-x}Al_xO$ (x = 0, 0.02, 0.04, 0.06) samples.

Nominal composition	D_{SEM} (μm)
ZnO	11.2
$Zn_{0.98}Al_{0.02}O$	0.9
$Zn_{0.96}Al_{0.04}O$	1.1
$Zn_{0.94}Al_{0.06}O$	1.1

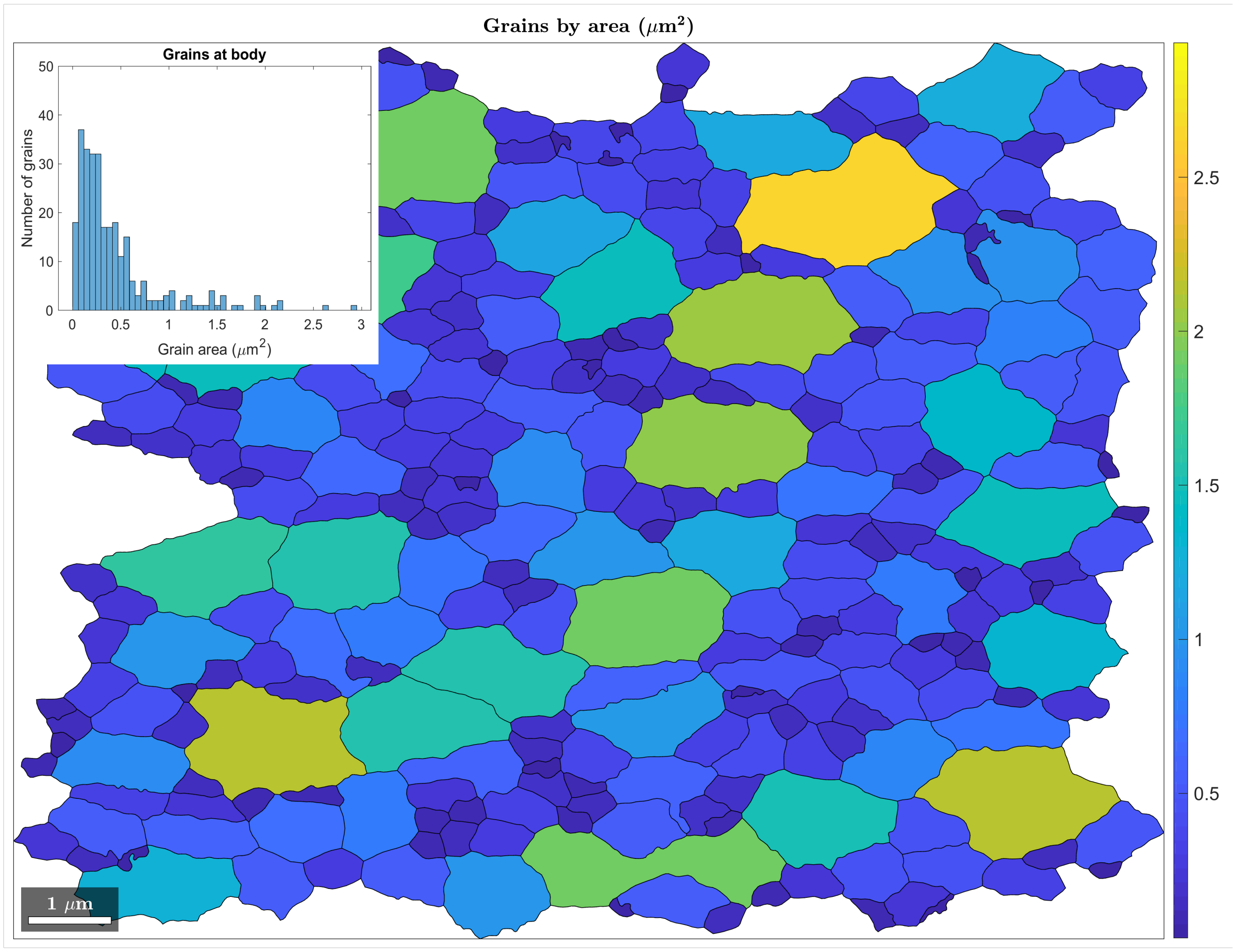

Figure S4: Grain size area distributions calculated from EBSD analysis for $Zn_{0.96}Al_{0.04}O$.

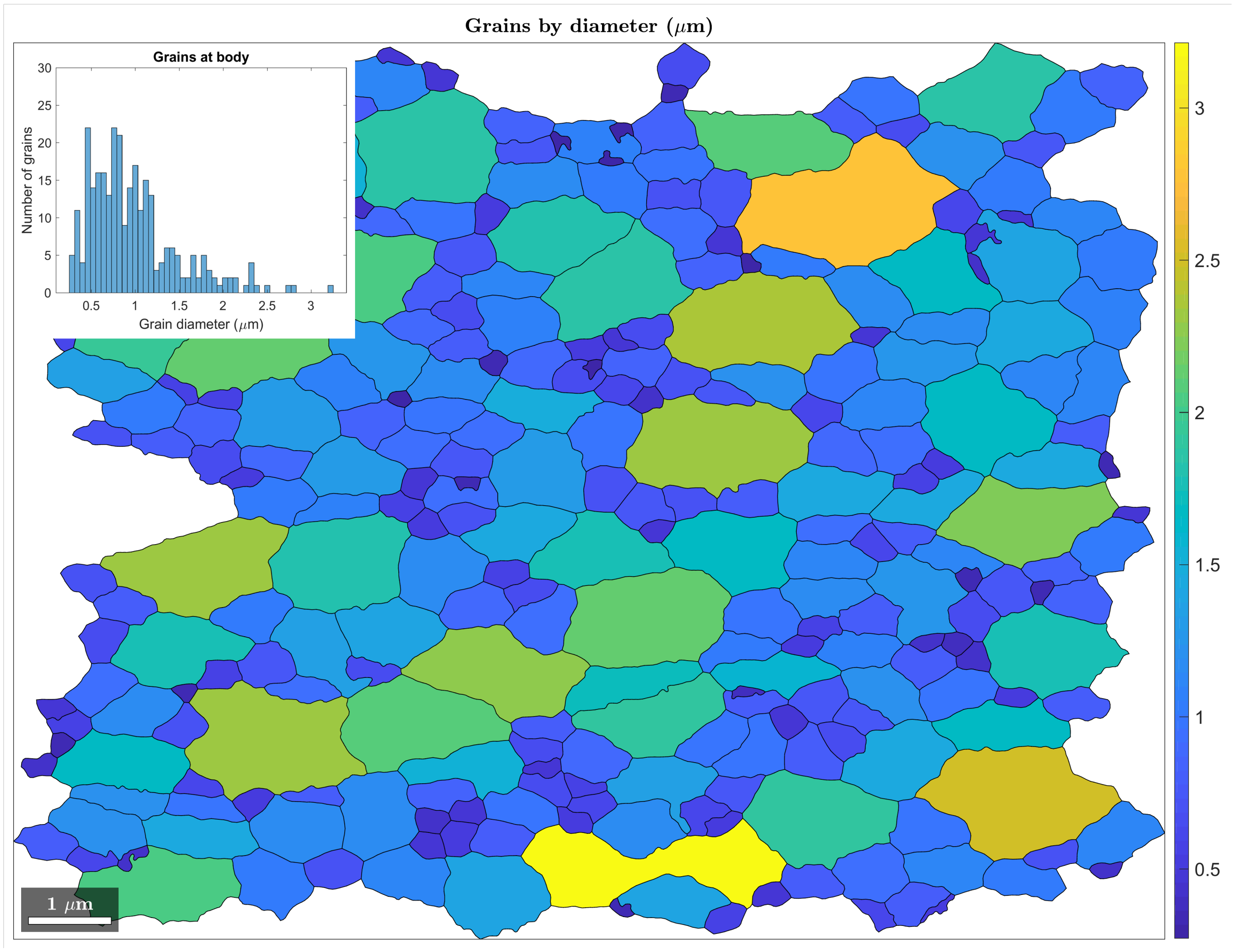

Figure S5: Grain size diameter distributions calculated from EBSD analysis for $Zn_{0.96}Al_{0.04}O$.

Single parabolic band model calculations

The experimental transport data can be analyzed using a single parabolic band (SPB) model with the relaxation time approximation.[1] The following equations are applicable for a single scattering mechanism, where the energy dependence of the carrier relaxation time can be expressed by a simple power-law $\tau = \tau_0 E^{\lambda}$, with λ being the scattering parameter.[2] The Hall factor r_{H}, given by:

$$r_H = \frac{3}{2} F_{\frac{1}{2}}(\eta) \frac{\left(\frac{3}{2}+2\lambda\right) F_{2\lambda+\frac{1}{2}}(\eta)}{\left(\frac{3}{2}+\lambda\right)^2 F^2_{\lambda+\frac{1}{2}}(\eta)}, \tag{1}$$

with the j-th order Fermi integrals, $F_j(\eta)$ defined by

$$F_j(\eta) = \int_0^{\infty} \frac{\varepsilon^j}{1+e^{\varepsilon-\eta}} d\varepsilon, \tag{2}$$

where ε is the reduced carrier energy, η is the reduced electrochemical potential related to Fermi energy via

$$\eta = \frac{E_F - E_c}{k_B T}. \tag{3}$$

where E_F is the Fermi level, E_c is the conduction band edge, and k_B is the Boltzmann constant. Reduced chemical potential can be calculated from Seebeck coefficient by the following equation:

$$\alpha = \frac{k_B}{e}\left[\frac{\left(\lambda+\frac{5}{2}\right) F_{\lambda+\frac{3}{2}}(\eta)}{\left(\lambda+\frac{3}{2}\right) F_{\lambda+\frac{1}{2}}(\eta)} - \eta\right], \tag{4}$$

where e is the electron charge. Lorenz number can be calculated as follows

$$L(\eta) = \pm\left(\frac{k_B}{e}\right)^2 \left(\frac{\left(\lambda+\frac{7}{2}\right) F_{\lambda+\frac{5}{2}}(\eta)}{\left(\lambda+\frac{3}{2}\right) F_{\lambda+\frac{1}{2}}(\eta)} - \left[\frac{\left(\lambda+\frac{5}{2}\right) F_{\lambda+\frac{3}{2}}(\eta)}{\left(\lambda+\frac{3}{2}\right) F_{\lambda+\frac{1}{2}}(\eta)}\right]^2\right). \tag{5}$$

Effective mass estimated by

$$m_d^* = \frac{h^2}{2 k_B T m_e}\left(\frac{n}{4\pi F_{1/2}(\eta)}\right)^{2/3}, \tag{6}$$

where m_d^* is the density-of-states effective mass, the chemical carrier concentration $n = n_H r_H$ (with n_H representing the Hall charge carrier concentration), h is the Planck's constant, and m_e the electron mass. Calculated parameters are presented in Table S3.

Table S3: Room-temperature Hall constant r_H, calculated chemical potential η, Lorenz number L, and effective mass m_d^* for $Zn_{1-x}Al_xO$ samples (x = 0, 0.02, 0.04, 0.06)

Nominal composition	r_H	η	L (V^2 K^{-2})	m_d^* (m_e)
ZnO	1.144	-0.3737	1.59	0.4
$Zn_{0.98}Al_{0.02}O$	1.084	1.7430	1.83	0.87
$Zn_{0.96}Al_{0.04}O$	1.084	1.7180	1.82	1.18
$Zn_{0.94}Al_{0.06}O$	1.067	2.3945	1.91	0.62

Low-temperature resistivity

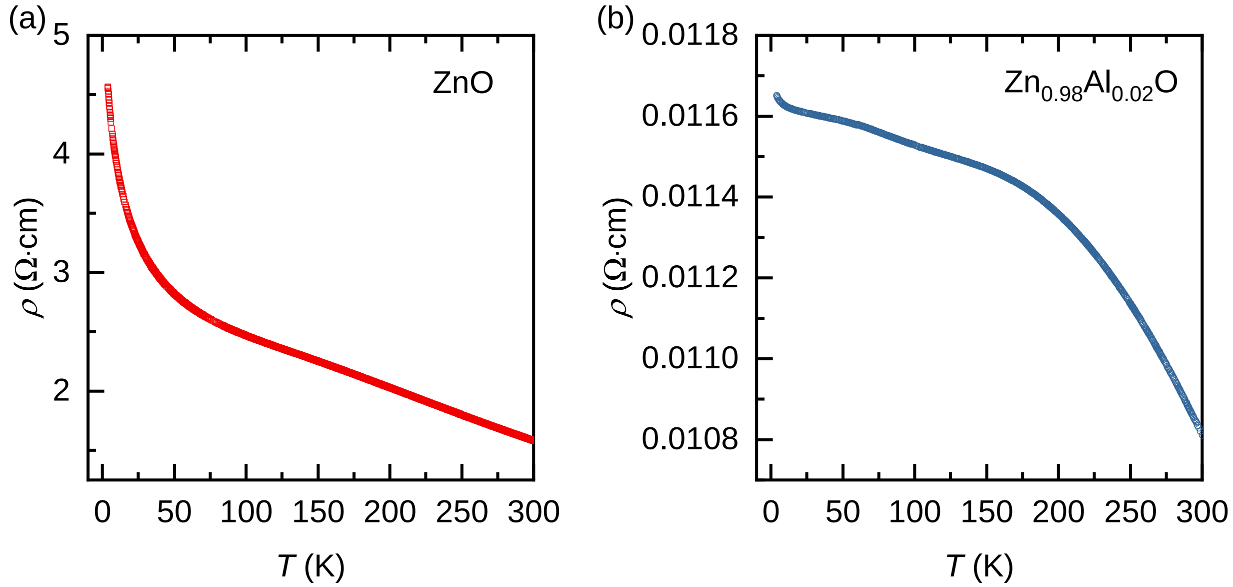

Figure S6: The experimental temperature dependence of resistivity for (a) ZnO and (b) $Zn_{0.98}Al_{0.02}O$ samples.

Hall effect

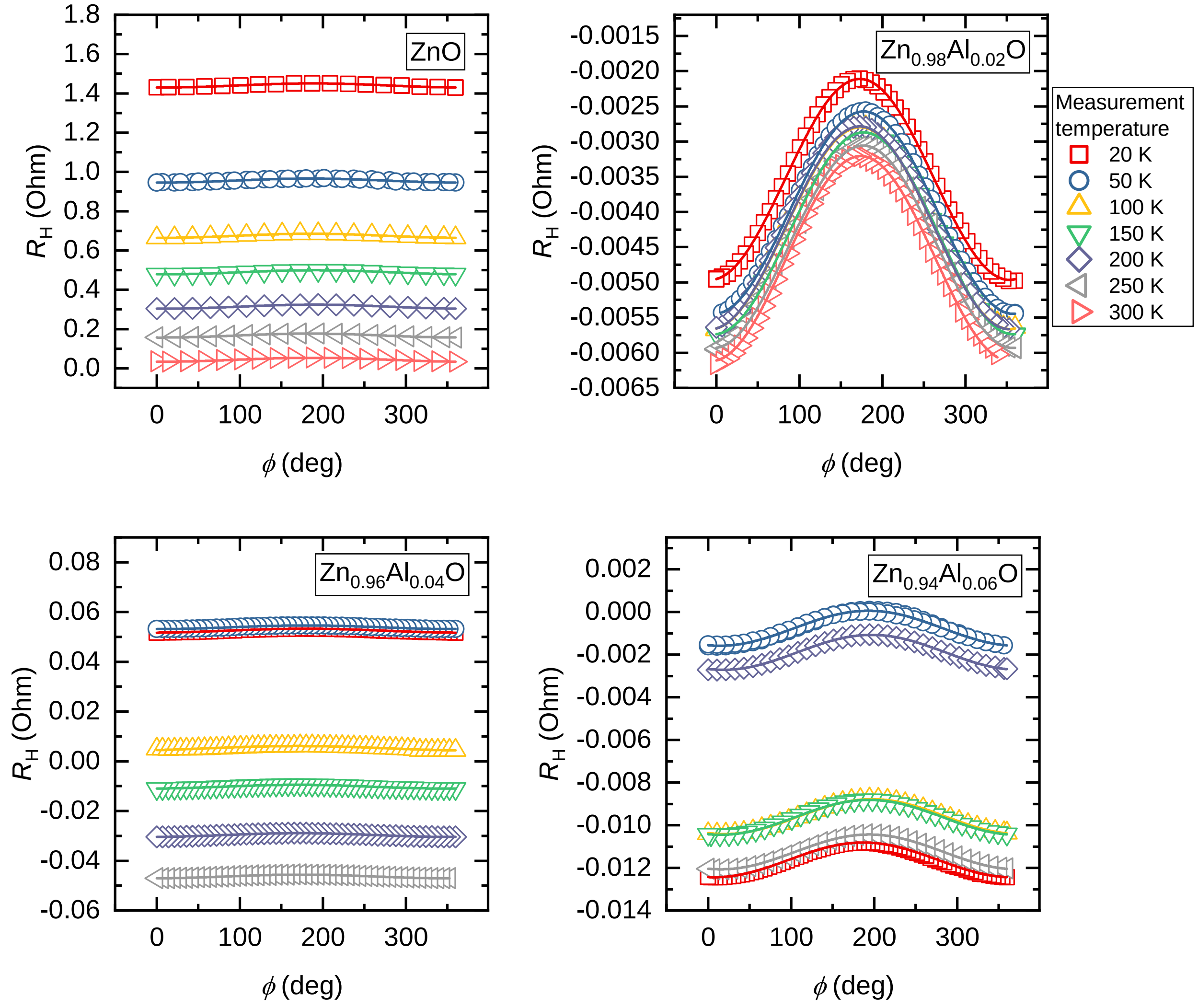

Figure S7: The experimental angular dependence of Hall resistivity (empty symbols) and its cosine fit (solid lines) for the $Zn_{1-x}Al_xO$ (x = 0, 0.02, 0.04, 0.06) samples.

The $\rho_H(\phi)$ data were produced by varying the angle between the normal to the plane of the sample n and the magnetic field H, resulting in a change in the scalar product (n, H). This modulation of the Hall signal follows a harmonic cosine law given by $\rho_H(\phi) = \rho_{H0} + \rho_{H1} \cos\phi$, where ρ_{H0} is the constant bias term, and ρ_{H1} is the main component of the Hall signal. The amplitude of the harmonic term, ρ_{H1}, was utilized to calculate the Hall coefficient $r_H = \rho_{H1}/H$. The Hall carrier concentration n_{H} and Hall carrier mobility μ_{H} were measured from 20 K to 300 K for the $Zn_{1-x}Al_xO$ (x = 0, 0.02, 0.04, 0.06) samples, as presented in Table S4.

Table S4: Temperature dependence of Hall carrier concentration n_{H}, and Hall carrier mobility μ_{H}.

Nominal composition	T (K)	n_{H} (10^{18} cm^{-3})	μ_{H} (cm^2 V^{-1} s^{-1})
ZnO	20	3.0	0.61
	50	3.0	0.73
	100	3.0	0.85
	150	3.0	0.93
	200	3.0	1.02
	250	3.1	1.13
	300	3.1	1.26
$Zn_{0.98}Al_{0.02}O$	20	48.2	11.16
	50	48.2	11.18
	100	48.2	11.24
	150	48.5	11.22
	200	48.5	11.33
	250	48.2	11.64
	300	47.5	12.15
$Zn_{0.96}Al_{0.04}O$	20	69.4	9.86
	50	70.3	10.03
	100	70.9	10.18
	150	71.4	10.29
	200	71.6	10.51
	250	72.0	10.55
	300	72.0	10.58
$Zn_{0.94}Al_{0.06}O$	20	39.7	7.89
	50	39.3	7.97
	100	39.4	7.99
	150	39.4	8.01
	200	39.3	8.03
	250	39.5	8.15
	300	39.2	8.39

Temperature dependence of Hall mobility

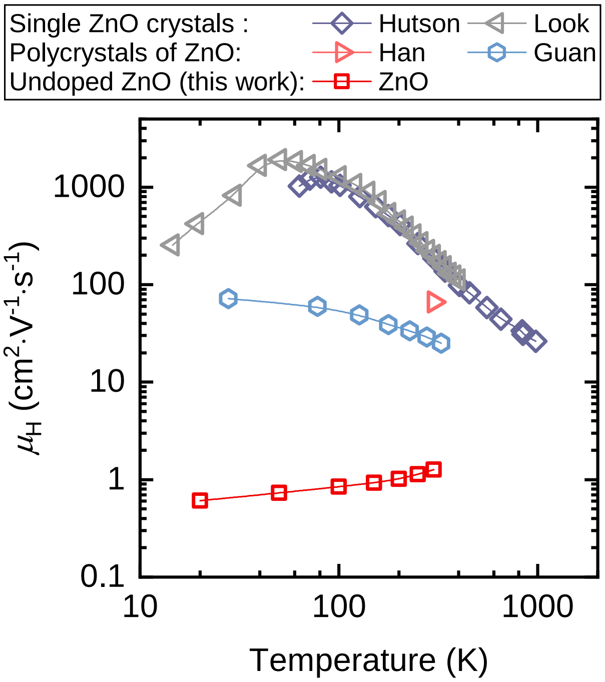

Figure S8: Temperature-dependent Hall mobility of our undoped ZnO sample compared to data from literature.[3–6]

Thermally activated transport

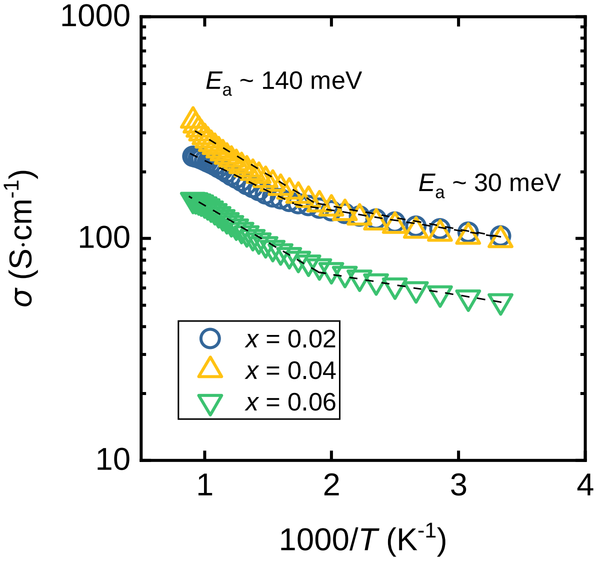

Figure S9: Thermally activated electrical conductivity in $Zn_{1-x}Al_xO$ (x = 0.02, 0.04, 0.06) samples.

Analysis of lattice thermal conductivity

The experimental data of κ_L were fitted and analyzed by the Debye-Callaway[7] model modified by Glassbrenner and Slack[8] for high temperatures above the Debye temperature ($T \gg \theta_D$). In the model by Glassbrenner and Slack the lattice thermal conductivity is given by

$$\kappa_L = \frac{k_B}{2\pi^2 \upsilon_a} \cdot \left(\frac{k_B T}{\hbar}\right)^3 \cdot \int_0^{\theta_D/T} \tau_c x^2 dx, \tag{7}$$

where υ_a is the average velocity of sound determined as

$$\upsilon_a = \left[\frac{1}{3}\left(\frac{1}{\upsilon_l^3} + \frac{2}{\upsilon_t^3}\right)\right]^{-1/3}, \tag{8}$$

with υ_t and υ_l being the transverse and longitudinal sound velocities ($2750\,\mathrm{m\,s^{-1}}$ and $5940\,\mathrm{m\,s^{-1}}$,[9] respectively), θ_D is the Debye temperature, $x = \hbar\omega/k_B T$, ω is the phonon frequency and τ_c is the total phonon relaxation time. The overall relaxation rate τ_c^{-1} can be determined by combining the various scattering processes based on Matthiessen's rule. For simplicity, we considered mainly phonon-phonon and point defect scattering, which are the most important contributions at high temperatures.

Thus, τ_c^{-1} is expressed as

$$\tau_c^{-1} = \sum_\imath \tau_\imath^{-1} = \tau_U^{-1} + \tau_{PD}^{-1} \tag{9}$$

where τ_U and τ_{PD}, are the relaxation times for Umklapp scattering and point defect scattering. Umklapp relaxation rate is defined as:

$$\tau_U^{-1} = \left(B_U T + B_H T^2\right)\omega^2 \tag{10}$$

where B_U and B_H are frequency and temperature independent coefficients, with B_H being a higher order four-scattering parameter.[8] Point defect term is expressed as:

$$\tau_{PD}^{-1} = A\omega^4 = \frac{V_a}{4\pi\upsilon_a^3}\Gamma\omega^4 \tag{11}$$

where V_a is the average atomic volume, Γ is the disorder scattering parameter.[10] According to Abeles and Slack the disorder scattering parameter Γ includes both, the strain field Γ_S and the mass fluctuation scattering Γ_M, and can be calculated by as $\Gamma = \Gamma_M + \Gamma_S$.

$$\Gamma_M = \frac{\sum_{i=1}^{n} c_i \left(\frac{\overline{M}_i}{\overline{\overline{M}}}\right)^2 f_i^1 f_i^2 \left(\frac{\mathrm{M}_i^1 - \mathrm{M}_i^2}{\overline{M}_i}\right)^2}{\sum_{i=1}^{n} c_i} \tag{12}$$

$$\Gamma_S = \frac{\sum_{i=1}^{n} c_i \left(\frac{\overline{M}_i}{\overline{\overline{M}}}\right)^2 f_i^1 f_i^2 \varepsilon_i \left(\frac{\mathrm{r}_i^1 - \mathrm{r}_i^2}{\overline{r}_i}\right)^2}{\sum_{i=1}^{n} c_i} \tag{13}$$

where n is the number of different crystallographic sublattice types in the lattice and c_i is the relative degeneracy of the respective sites. In the pure ZnO, $n = 2$, $c_i = 2$, $\overline{\overline{M}}$ is the average atomic mass, f_i^k is the fractional occupation of the k-th atom on the i-th site, M_i^k and r_i^k are the atomic mass and radius of the k-th atom, ε_i is a function of the Grüneisen parameter, γ, which characterizes the anharmonicity of the lattice.[11] $\overline{M}_i$ and $\overline{r}_i$ are the average atomic mass and radius on the i-th site, respectively:

$$\overline{M}_i = \sum_k f_i^k M_i^k, \overline{r}_i = \sum_k f_i^k r_i^k \tag{14}$$

For the strain field fluctuation, ε is directly estimated by:

$$\varepsilon = \left[\sqrt{2}\frac{\psi}{1+\psi}\left(-4 - 6.4\gamma\right)\right]^2 \tag{15}$$

here ψ determined as

$$\psi = \frac{1 - \upsilon_p}{2\left(1 - 2\upsilon_p\right)} \tag{16}$$

where υ_p is the Poison ratio:

$$\upsilon_p = \frac{1 - 2\left(\upsilon_t - \upsilon_p\right)^2}{2 - 2\left(\upsilon_t - \upsilon_p\right)^2} \tag{17}$$

and

$$\gamma = \frac{3}{2}\left(\frac{1 + \upsilon_p}{2 - 3\upsilon_p}\right) \tag{18}$$

Obtained results are shown in Table S5

Table S5: Thermal conductivity fitting parameters

Nominal composition	$M_{unitcell}$ (g/mol)	$V_{unitcell}$ (Å^3)	Γ_m	Γ_s	Γ
ZnO	162.758	47.6377	-	-	-
$Zn_{0.98}Al_{0.02}O$	161.222	47.5589	0.0022	0.27	0.272
$Zn_{0.96}Al_{0.04}O$	159.686	47.5616	0.0044	0.534	0.538
$Zn_{0.94}Al_{0.06}O$	158.15	47.5713	0.0067	0.792	0.799

References

(1) Kireev, P. S. *Semiconductor Physics, 2nd ed.*; Mir Publishers, 1978; Vol. 1.

(2) Fistul, V. I. *Heavily doped semiconductors*; Springer New York, 1969; Vol. 1.

(3) Hutson, A. Electronic properties of ZnO. *Journal of Physics and Chemistry of Solids* **1959**, *8*, 467–472.

(4) Look, D. C.; Reynolds, D. C.; Sizelove, J.; Jones, R.; Litton, C. W.; Cantwell, G.; Harsch, W. Electrical properties of bulk ZnO. *Solid state communications* **1998**, *105*, 399–401.

(5) Han, L. High Temperature Thermoelectric Properties of ZnO Based Materials. Ph.D. thesis, 2014.

(6) Guan, W.; Zhang, L.; Wang, C.; Wang, Y. Theoretical and experimental investigations of the thermoelectric properties of Al-, Bi-and Sn-doped ZnO. *Materials Science in Semiconductor Processing* **2017**, *66*, 247–252.

(7) Tritt, T. M. *Thermal conductivity: theory, properties, and applications*; Springer Science & Business Media, 2005.

(8) Glassbrenner, C. J.; Slack, G. A. Thermal conductivity of silicon and germanium from 3 K to the melting point. *Physical review* **1964**, *134*, A1058.

(9) Han, L. High Temperature Thermoelectric Properties of ZnO Based Materials. Ph.D. thesis, 2014.

(10) Ren, G.-K.; Lan, J.-L.; Ventura, K. J.; Tan, X.; Lin, Y.-H.; Nan, C.-W. Contribution of point defects and nano-grains to thermal transport behaviours of oxide-based thermoelectrics. *npj Computational Materials* **2016**, *2*, 1–9.

(11) Abeles, B. Lattice thermal conductivity of disordered semiconductor alloys at high temperatures. *Physical Review* **1963**, *131*, 1906.
